# Modulatory effects of platelet-rich plasma on viral kinetics of BoAHV-1.1, BoGHV-4, and BVDV in bovine cell cultures: A proof-of-concept study

**DOI:** 10.1016/j.virusres.2025.199653

**Published:** 2025-10-31

**Authors:** Valentina Andreoli, Sofia Lopez, Santiago Germán Delgado, Sandra Elizabeth Pérez, Susana Beatriz Pereyra, Erika Analía Gonzalez Altamiranda, Florencia Romeo, Stefano Grolli, Andrea Elizabeth Verna

**Affiliations:** a Department of Veterinary Medical Science, University of Parma, 43121 Parma, Italy; b Agencia Nacional de Promoción Científica y Tecnológica (ANPCyT), Godoy Cruz 2370 C1425FQD Buenos Aires, Argentina; c Universidad Nacional de Mar del Plata, Facultad de Ciencias Agrarias, Argentina; d Centro de Investigación Veterinaria de Tandil (CIVETAN)-CONICET. Facultad de Ciencias Veterinarias, Universidad Nacional del Centro de la Provincia de Buenos Aires, Argentina; e Instituto Nacional de Tecnología Agropecuaria, Instituto de Innovación para la Producción Agropecuaria y El Desarrollo Sostenible (IPADS, INTA-CONICET) Ruta 226, km 73.5, Balcarce CC7620, Buenos Aires, Argentina; f Consejo Nacional de Investigaciones Científicas y Técnicas (CONICET) , Argentina

**Keywords:** Cattle, Platelet-rich plasma, PRP, BoAHV-1.1, BoGHV-4, BVDV, BESc

## Abstract

Reproductive viral diseases caused by Bovine alphaherpesvirus 1.1 (BoAHV-1.1), Bovine gam-maherpesvirus 4 (BoGHV-4), and Bovine viral diarrhoea virus (BVDV) impose a substantial economic burden on the cattle industry, primarily through infertility, abortion, and impaired reproductive performance. Owing to the limited efficacy of current antiviral strategies, this study evaluated the *in vitro* effects of platelet-rich plasma (PRP), at concentrations of 5 % and 10 %, on the replication kinetics of these viruses in Madin-Darby bovine kidney (MDBK) cells and primary bovine endometrial stromal cells (BESc). PRP modulated viral replication in a virus-, cell type-, and dose-related manner. In BoAHV-1.1-infected MDBK cells, 10 % PRP reduced extracellular titres but increases intracellular accumulation, suggesting interference with viral egress. In BESc, both intra- and extracellular titres decreased, consistent with a broader antiviral effect. For BoGHV-4 and BVDV, PRP induced variable and time-dependent responses across cell types. These results demonstrate that PRP can influence bovine viral replication dynamics *in vitro* and support further investigations into its mechanistic basis and *in vivo* therapeutic potential.

## Highlights

• PRP modulates BoAHV-1.1, BoGHV-4, and BVDV replication in MDBK and BESc cells.
• 10 % PRP reduces extracellular BoAHV-1.1 titers in both MDBK and BESc cultures.
• BoGHV-4 shows cell-dependent responses to PRP, with selective viral inhibition.
• PRP emerges as a promising antiviral tool for bovine endometrial viral infections.

## Graphical abstract

**Figure:**
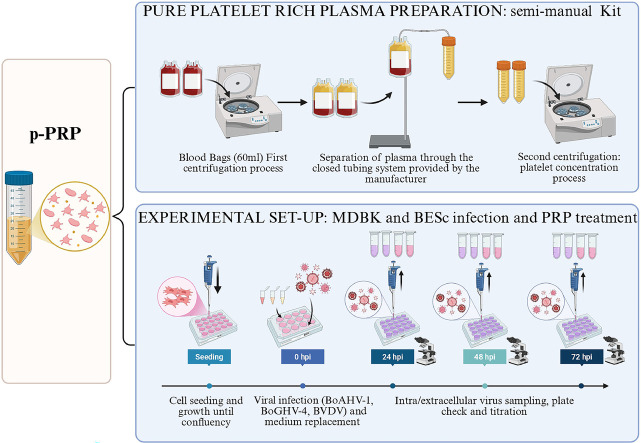

## 1 Introduction

Livestock production is a key component of Argentina’s economy, with both beef and dairy sectors contributing substantially to national income (Arelovich et al., 2011; Kenneth, 2023; Lazzarini et al., 2019). In dairy herds, reproductive disorders remain a major cause of economic loss due to reduced fertility, increased calving intervals, and abortions (Giuliodori et al., 2013; Plöntzke et al., 2010). Among infectious agents, viruses such as bovine alphaherpesvirus 1.1 (BoAHV-1.1), bovine gammaherpesvirus 4 (BoGHV-4), and bovine viral diarrhea virus (BVDV) are key contributors to bovine reproductive disease worldwide (Atasever et al., 2023; Sheldon et al., 2009). BoAHV-1.1 (Spilki et al., 2004)) and BVDV are endemic in Argentina, whereas BoGHV-4 is emerging (Morán et al., 2015; Spilki et al., 2004). Notably, BVDV can establish persistent infections following fetal exposure in utero (Ridpath, 2010) further complicating eradication efforts. The selection of the viruses for this study is based on their relevance as major pathogens in reproductive diseases affecting cattle fertility (Atasever et al., 2023; Henderson and Caldow, 2023; Wathes et al., 2020). Despite the availability of vaccines for BoAHV-1.1 and BVDV (Brock, 2003; Raaperi et al., 2014; Zhang et al., 2024), no antiviral therapy or vaccine exists for BoGHV-4, and the most effective live-attenuated vaccines remain restricted in several countries, including Argentina (Campero et al., 2017). Consequently, treatment of viral reproductive disorders relies mainly on supportive and anti-inflammatory measures (Radostits and Done, 2007), highlighting the need for alternative, host-targeted strategies. Platelet-rich plasma (PRP) is a biological product obtained through a double centrifugation protocol of whole peripheral blood (Everts et al., 2020). This product is generally characterized by platelet concentrations three to ten times higher than baseline, with 1 × 10⁹ platelets/mL widely regarded as a benchmark for high-quality preparations (DeLong et al., 2012; Roukis et al., 2006; Sethi et al., 2021). PRP has emerged as a biological product with regenerative, anti-inflammatory, and antimicrobial properties (Li et al., 2019; Masuki et al., 2016). Upon activation, platelets release numerous bioactive molecules (Giusti et al., 2020), including growth factors, chemokines, and antimicrobial peptides such as platelet factor 4 (PF4) and defensins (Arora et al., 2016; El-Sharkawy et al., 2007; Manne et al., 2017; Rossaint et al., 2018), several of which have demonstrated direct antiviral activity against viruses including HIV-1, Dengue, and Japanese encephalitis virus (Auerbach et al., 2012; Ojha et al., 2019; Pei et al., 2022). In cattle, this product has been successfully applied to reproductive disorders such as endometritis and repeat breeder syndrome, primarily due to its immunomodulatory and tissue-repairing effects (Cremonesi et al., 2020a, 2020b; Lange-Consiglio et al., 2023, 2015; Marini et al., 2016). However, its potential antiviral role in the bovine reproductive tract remains largely unexplored. Our previous work demonstrated that PRP secretions can modulate inflammatory and antiviral gene expression in bovine endometrial cells challenged with BoGHV-4 and bacterial lipopolysaccharide (López et al., 2025), supporting the hypothesis that PRP may indirectly limit viral replication by modifying the host cellular environment. Based on this observations, we hypothesise that bovine PRP exerts a modulatory effect on viral replication dynamics through alterations in innate immune signaling and cellular microenvironment. To test this hypothesis, the present study investigates, for the first time, the *in vitro* effects of PRP (5 % and 10 %) on the replication kinetics of BoAHV-1.1, BoGHV-4, and BVDV in Madin-Darby bovine kidney (MDBK) cells and bovine endometrial stromal cells (BESc).

## 2 Materials and methods

### 2.1 MDBK cell culture

Madin-Darby bovine kidney (MDBK) cells from the American Type Culture Collection (ATCC, Rockville, MD, USA) were used for *in vitro* assays. The cell line was cultured in minimal essential medium with Earles' salts (MEM-E, Gibco; Thermo Fish-er Scientific, Carlsbad, CA, USA) containing 10 % fetal bovine serum (FBS) (Bioser, Buenos Aires, Argentina, USA), an antibiotic-antimitotic solution (Gibco, Langley, OK, USA) that included 100 U of penicillin G, 100 μg of streptomycin sulphate, and 0.025 μg of amphotericin B per ml. Cells were incubated at 37 °C with 5 % CO2 in a humidified incubator and the culture medium was changed every 48 h and left until confluence was reached. FBS was analysed by qPCR-based screening for BoAHV-1.1, BoGHV-4, and BVDV, to exclude a contamination of the serum.

### 2.2 Bovine endometrial stromal cells (BESc): isolation and primary culture

Primary endometrial cells were procured from bovine uteri selected from healthy donors at the Balcarce slaughterhouse (Balcarce, Buenos Aires, Argentina). The uteri were transported under sterile conditions within one hour to the cell culture laboratory, where they underwent screening for viral and bacterial contamination. To ensure that all cell cultures were free of endemic bovine viruses, total RNA/DNA was extracted using the Viral DNA/RNA Purification Kit (Roche), and the presence of BoAHV-1.1, BoGHV-4, and BVDV was assessed by quantitative PCR (qPCR), following the protocol described by Romeo et al. (2022) (Romeo et al., 2022).This molecular screening is routinely applied in our laboratory as a standard quality control step prior to experimental use, while bacterial sterility was confirmed through microbiological procedures. The uteri were subjected to external disinfection with ethanol and phosphate-buffered saline (PBS) washes prior to tissue processing. Following disinfection, tissue layers from the uterine horn containing a follicle were carefully dissected using sterile instruments (Romeo et al., 2024; Tebaldi et al., 2016). The superficial endometrial layer was excised, fragmented, and subjected to enzymatic digestion with 0.1 % (w/v) collagenase type IV (GIBCO) in MEM-E at 37 °C for one hour with constant agitation. Digestion was halted with MEM-E supplemented with 10 % FBS, and the resulting suspension was filtered and centrifuged. The resultant pellet, rich in BESc, was suspended in MEM-E supplemented with 10 % FBS and antibiotics (as described in Section 2.1) before being seeded into 25 cm² culture flasks. Cells were incubated at 37 °C in a 5 % CO₂ atmosphere, with media changes every 48 h. Expansion continued until passage 3, at which point the cells were cryopreserved for subsequent experimental use, ensuring all analyses were conducted on populations below passage 4. Our decision to utilize cells at passage 3 (P3) was informed by both our prior observations and supporting literature demonstrating that early passages (P2–P4) preserve optimal morphological characteristics and functional responsiveness in bovine endometrial stromal cells (Romeo et al., 2024; Tebaldi et al., 2016). Throughout our standardized isolation and culture protocols, we meticulously monitored cellular morphology from passage 0 (P0) through passage 3 (P3). The cells consistently exhibited their characteristic fibroblast-like morphology, maintained strong adherence, and demonstrated robust proliferative capacity across these passages, without evidence of morphological deviation, senescence, or spontaneous differentiation

### 2.3 Virus strains

In the study three distinct viral strains were employed to assess the efficacy of the product. These included:

- *Varicellovirus bovinealpha-1* BoAHV-1.1 Los Angeles 38 (LA38) strain: A well-characterized reference strain
- BoGHV-4 strain 07/435: Field isolate from cervicovaginal mucus of a cow that has suffered an abortion.
- Bovine Viral Diarrhea Virus (BVDV) strain NADL VS 145 genotype 1.

Each virus was propagated separately in MDBK cells using T-25 flasks (Greiner Bio-One, Nümbrecht, Germany) at a density of 1 × 10^5 cells/mL. The cell cultures were maintained for 48 h to prepare the viral stocks for subsequent *in vitro* assays. The culture medium used is described in Section 2.1. Supernatants were harvested and stored at − 80 °C until use.

### 2.4 Platelet- rich plasma preparation

PRP was prepared from whole blood of donor cows belonging to the INTA Balcarce institutional herd, following the CICUAE recommendations (232/2021). To ensure consistency and minimize inter-donor variability in this initial screening assay of viral kinetics, all PRP used in this study was obtained from a single donor animal. PRP preparation was carried out using a semi-automated, closed and sterilized disposable kit (Ematik® Kit Semi-manuale, Prometheus Srl, Parma, PR, Italy) as described by Palagiano and colleagues (Palagiano et al., 2023). Briefly, blood was collected from donor animals using a 60 mL syringe containing 5 mL of 4 % sodium citrate as an anticoagulant (provided). For each donor, a maximum of 120 mL (two 60 mL syringes) of blood was collected. PRP was subsequently prepared using the closed tube system provided in the kit, following the manufacturer's protocols. After two centrifugation steps, the PRP was resuspended in 6 mL according to the manufacturer's guidelines and extracted from the kit container using a 10 mL luer lock syringe. PRP was aliquoted in 1.5 ml tubes and frozen once at –20 °C until use. No cryoprotectants were added. This approach follows validated PRP protocols showing preserved platelet activity and growth factor release after freezing process (Hosseini et al., 2023; Meftahpour et al., 2021). Prior to freezing, a small aliquot of the PRP was taken for manual platelet counting. The PRP preparations achieved a final platelet concentrations of 1 × 10^9^ ± 0.2 × 10^9^ platelets/mL.

### 2.5 Viral infection and PRP treatment

To investigate the role of PRP in viral replication, MDBK cells and BESc were cultured in 12-well plates (Greiner Bio-One, Nümbrecht, Germany) at a density of 7.5 × 10^5 cells/mL in MEM-E supplemented with 10 % FBS (Group 1 or control group), 5 % PRP (Group 2), or 10 % PRP (Group 3). The cells were incubated at 37 °C in a 5 % CO2 atmosphere. Each treatment group was performed in triplicate. When the cell monolayers reached approximately 90 % confluence, they were infected with 2000 TCID₅₀ of each virus, as determined by Reed–Muench endpoint dilution (Reed and Muench, 1938). Following a 2-hour incubation period, the supernatant was removed and replaced with fresh medium (MEM-E with either FBS or PRP). Non-infected wells were included as internal controls for both the cell line and the primary culture. The use of the MDBK cell line facilitated a controlled and comparable experiment, enabling the evaluation of PRP efficacy against the different viral strains in comparison to FBS. To minimize experimental variability and ensure consistency in PRP composition, a single PRP preparation obtained from one clinically healthy, non-pregnant donor heifer was used throughout the study. All conditions were tested in technical triplicates under uniform *in vitro* settings.

### 2.6 Replication kinetics of boahv-1.1, boghv-4 and BVDV

The replication kinetics of BoAHV-1.1, BoGHV-4, and BVDV strains were assessed at both intracellular and extracellular levels in MDBK and BESc cells. For intracellular titration, infected cells were frozen at −80 °C and subsequently thawed to promote the release of viral particles into the medium. Samples from both intracellular and extracellular sources were collected at 24, 48, and 72 h post-infection (hpi) for virus titration using the endpoint titration method with both cell types cultured in 96-well microtiter plates (Greiner Bio-One, Nümbrecht, Germany). The presence of cytopathic effects (CPE) was monitored daily under a microscope. Viral titers were calculated at 72 hpi and expressed as TCID50/mL (Reed and Muench, 1938). Titers were independently assessed by three different investigators, and the values were averaged. Each assay was performed in triplicate, with appropriate negative controls (mock-infected cells) included.

### 2.6 Statistical analysis

Statistical analysis of viral kinetics was conducted using R studio (R core Team, 2022). For each combination between BoAHV-1.1, BoGHV-4, and BVDV strains and both, intracellular and extracellular level, viral replication data collected at different time points (24, 48 and 72 h) were analyzed to assess the effect of PRP on the replication dynamics of the three viruses in MDBK cells and BESc. Prior to performing the analysis of variance (ANOVA) and Least Significant Difference (LSD) post hoc tests, all datasets were assessed for compliance with statistical assumptions. Normality was evaluated using the Shapiro–Wilk test, and homogeneity of variance was assessed via Levene’s test. Only after confirming these assumptions were the ANOVA and LSD tests applied. Simple linear and quadratic regression models were used to fit the viral growth curves and estimate key parameters, including peak viral titers, replication rates, and time to peak. In all hypothesis tests performed, the significance level used was five percent (α=0.05). The results were visualized using graphical outputs generated with the **ggplot2** package to depict the impact of PRP on viral replication.

## 3 Results

### 3.1 Replication kinetics of boahv-1.1 in MDBK and BESc cells

The replication dynamics of BoAHV-1.1 were evaluated in both MDBK and BESc at intracellular and extracellular levels (**Tables S1–S2, Appendix A**).

**Fig. 1:**
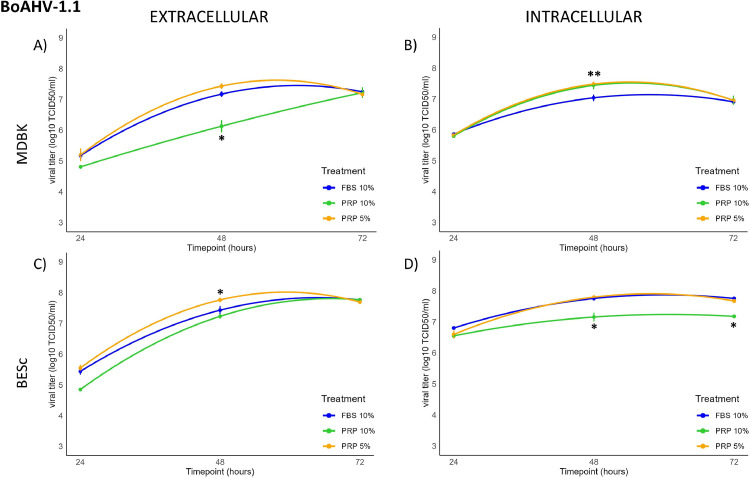
Extracellular and intracellular BoAHV-1.1 titers (log₁₀ TCID₅₀/ml) at 24, 48, and 72 h post-infection (hpi) in MDBK and BESc cultures. Panel A: extracellular titers in MDBK cells; Panel B: intracellular titers in MDBK cells; Panel C: extracellular titers in BESc; Panel D: intracellular titers in BESc.Values represent the means ± SD of three independent replicates. Asterisks (*) indicate significant differences (p < 0.01) between PRP (5 % or 10 %) and FBS (10 %) at each time point.

**Fig. 2:**
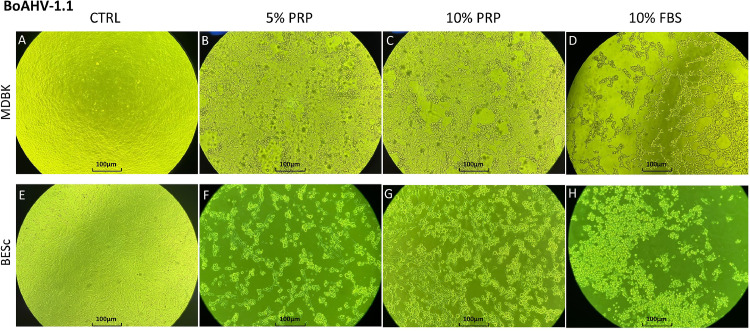
Cytopathic effect (CPE) of BoAHV-1.1 observed at 48 h post-infection (hpi) under different culture conditions in MDBK and BESc cells. Panels A–D correspond to MDBK cultures: A, uninfected control; B, 5 % PRP; C, 10 % PRP; D, 10 % FBS. Panels E–H correspond to BESc cultures: E, uninfected control; F, 5 % PRP; G, 10 % PRP; H, 10 % FBS. Images were captured at 10× magnification. The cytopathic effect is characterized by cell rounding and detachment, with more pronounced degeneration observed in FBS-treated groups compared with PRP-treated or control cultures.

In MDBK cells, significant changes (*p* < 0.01) were observed only intracellularly at 48 h post-infection (hpi), with a 0.5 log increase in viral titer detected under 5 % and 10 % PRP treatment. Intracellular viral kinetics followed a concave ascending quadratic model (Fig. 1A), reaching maximal titers at 48 hpi. Extracellularly (Fig. 1B), titers showed a similar concave trend with peaks at 48 hpi under 5 % PRP and 10 % FBS, whereas treatment with 10 % PRP produced a linearly ascending pattern up to 72 hpi, suggesting delayed viral release. Morphologically (Fig. 2A, B, C and D), MDBK monolayers in the 10 % FBS group were completely lysed at 48 hpi, while PRP-treated cultures maintained viable adherent layers.

In BESc cultures, extracellular viral titers, differed significantly as early as 24 hpi (*p* < 0.01), with the 10 % PRP treatment showing the lowest values. At 48 hpi, 5 % PRP yielded significantly higher extracellular titers than the other groups (*p* < 0.01), while no further differences were observed at 72 hpi. Intracellularly, no changes were detected at 24 hpi, but at both 48 and 72 hpi, the 10 % PRP treatment resulted in titers nearly 1 log₁₀ lower than other conditions (*p* < 0.001). Replication curves for BESc also fitted a concave quadratic model with peaks at 48 hpi, followed by a slight decline thereafter (Fig. 1C and D).

Microscopic examination (Fig. 2E, F, G and H) revealed marked cytopathic effects (CPE) under 10 % FBS, with cell detachment and death at 48 hpi, whereas PRP-treated wells displayed residual viable cells and reduced monolayer disruption. Together, these results suggest that PRP exerts cell type–dependent modulation of BoAHV-1.1 replication, enhancing intracellular accumulation and delaying viral egress in MDBK cells, while reducing both intra- and extracellular titers in BESc, indicative of a broader antiviral effect.

### 3.2 Replication kinetics of boghv-4 in MDBK and BESc cells

The replication kinetics of BoGHV-4 strain 07/435 were evaluated in both MDBK and BESc at intracellular and extracellular levels (**Tables S3–S4, Appendix A**).

**Fig. 3:**
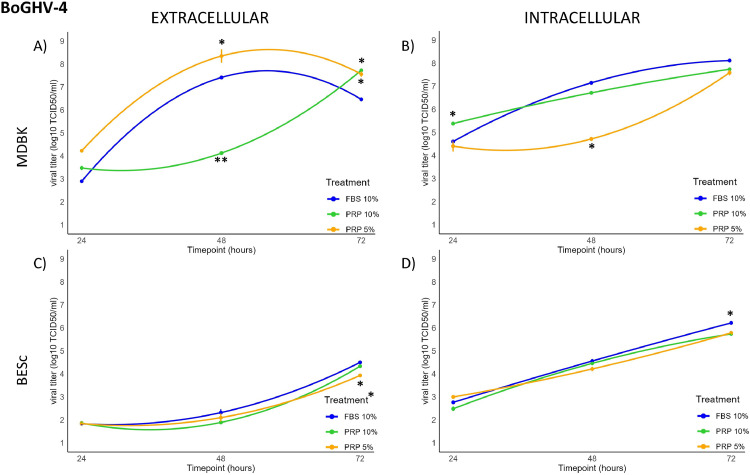
Fig. 3. Extracellular and intracellular BoGHV-4 titers (log₁₀ TCID₅₀/ml) at 24, 48, and 72 h post-infection (hpi) in MDBK and BESc cultures. Panel A: extracellular titers in MDBK cells; Panel B: intracellular titers in MDBK cells; Panel C: extracellular titers in BESc; Panel D: intracellular titers in BESc. Values represent the means ± SD of three independent replicates. Asterisks (*) indicate significant differences (p < 0.01 for MDBK; p < 0.05 for BESc) between PRP (5 % or 10 %) and FBS at each time point.

**Fig. 4:**
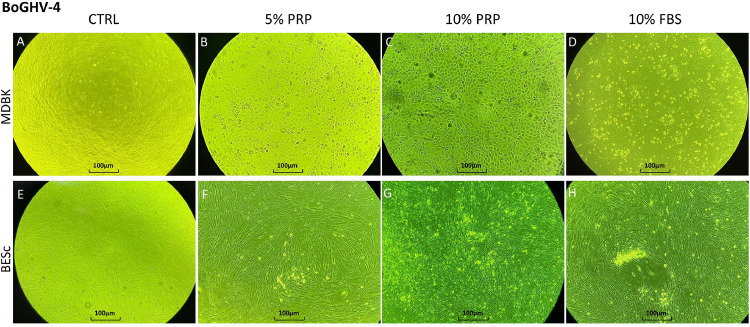
Cytopathic effect (CPE) of BoGHV-4 (strain 07/435) observed at 48 h post-infection (hpi) under different culture conditions in MDBK and BESc cells. Panels A–D correspond to MDBK cultures: A, uninfected control; B, 5 % PRP; C, 10 % PRP; D, 10 % FBS. Panels E–H correspond to BESc cultures: E, uninfected control; F, 5 % PRP; G, 10 % PRP; H, 10 % FBS. Images were captured at 40× magnification. The cytopathic effect is characterized by cell rounding and detachment, with more pronounced alterations observed in the 10 % FBS condition compared with PRP-treated and control groups.

In MDBK cells, extracellular viral titers showed an early increase (*p* < 0.01) at 24 hpi under 5 % PRP treatment, followed by a 1 log rise compared with the 10 % FBS control at 48 hpi. However, increasing PRP concentration to 10 % resulted in a marked 4-log reduction (*p* < 0.01). By 72 hpi, viral titers in PRP-treated cultures increased again by approximately 1 log relative to the control (*p* < 0.01). Intracellularly, a 1-log increase was detected at 24 hpi with 10 % PRP, but at 48 hpi the 5 % PRP condition showed a significant 3-log decrease (*p* < 0.01). No significant differences were observed among treatments at 72 hpi. The overall kinetics followed a concave quadratic trend (Fig. 3A and B), with extracellular titers ascending under 5 % PRP but declining intracellularly, while 10 % PRP induced the opposite pattern: reduced extracellular but elevated intracellular titers. Morphologically (Fig. 4A, B, Fig. 4, Fig. 4), MDBK monolayers exposed to PRP, particularly at 5 %, remained largely intact with limited detachment, whereas cultures treated with 10 % FBS displayed characteristic cytopathic “holes” indicating virus-induced damage.

In BESc cultures, BoGHV-4 replication exhibited a more uniform pattern with moderate modulation by PRP (Fig. 3C and D). No significant extracellular differences were detected at 24 or 48 hpi (*p* > 0.05). At 72 hpi, cells treated with 5 % PRP showed titers approximately 0.5 log₁₀ lower than the other treatments (*p* < 0.05). Intracellular titers followed a similar pattern: no significant changes were observed up to 48 hpi, but at 72 hpi cells supplemented with 10 % FBS exhibited a 0.8 log₁₀ increase compared to 10 % PRP (*p* < 0.01). As shown in Fig. 4E, F, Fig. 4, Fig. 4, BESc monolayers maintained structural integrity across all treatments, with only mild cytopathic alterations under FBS supplementation.

Overall, these results indicate that PRP modulates BoGHV-4 replication in a dose- and cell type–dependent manner. In MDBK cells, 5 % PRP transiently enhanced extracellular viral release, while higher PRP concentration (10 %) markedly suppressed it and increased intracellular accumulation, suggesting interference with viral egress. In BESc, both PRP concentrations produced mild inhibitory effects, particularly at later time points, indicating a more stable antiviral environment compared to the permissive MDBK cell line.

### 3.3 Replication kinetics of BVDV in MDBK and BESc cells

The replication kinetics of the BVDV NADL strain were assessed at both intracellular and extracellular levels in MDBK and BESc cells **(Tables S5–S6, Appendix A).**

**Fig. 5:**
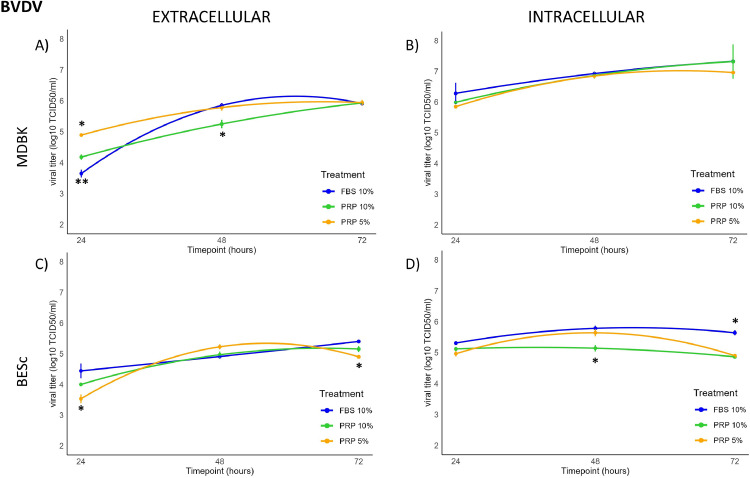
Extracellular and intracellular BVDV titers (log₁₀ TCID₅₀/ml) at 24, 48, and 72 h post-infection (hpi) in MDBK and BESc cultures. Panel A: extracellular titers in MDBK cells; Panel B: intracellular titers in MDBK cells; Panel C: extracellular titers in BESc; Panel D: intracellular titers in BESc. Values represent the means ± SEM of three independent replicates. Asterisks (*) indicate significant differences (p < 0.01) between PRP (5 % or 10 %) and FBS at each time point.

**Fig. 6:**
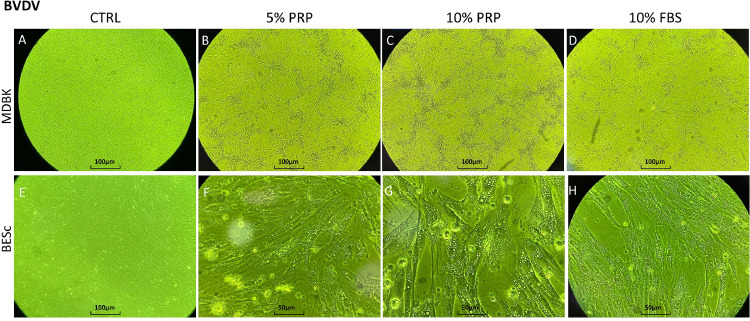
Cytopathic effect (CPE) of Bovine Viral Diarrhea Virus (BVDV, strain NADL) observed at 48 h post-infection (hpi) under different culture conditions in MDBK and BESc cells. Panels A–D correspond to MDBK cultures: A, uninfected control; B, 5 % PRP; C, 10 % PRP; D, 10 % FBS. Panels E–H correspond to BESc cultures: E, uninfected control; F, 5 % PRP; G, 10 % PRP; H, 10 % FBS. Images were captured at 40× magnification. The cytopathic effect is characterized by cell rounding and detachment, with more severe degeneration observed in the 10 % FBS condition compared with PRP-treated and control groups.

In MDBK cells, extracellular viral titers increased significantly (*p* < 0.01) by approximately 1 log₁₀ at 24 h post-infection (hpi) in the presence of PRP. Among treatments, 5 % PRP induced the highest extracellular titer, whereas at 48 hpi, cultures exposed to 10 % PRP displayed significantly reduced viral loads (*p* < 0.01). Intracellular titers did not differ significantly across treatments during the early infection phase, but all conditions reached their maximum viral accumulation at 72 hpi, showing a concave ascending quadratic trend (Fig. 5A and B). This temporal pattern suggests that PRP mainly influences early replication and release rather than the late-stage accumulation of virions. Microscopic examination at 48 hpi (Fig. 6A, B, Fig. 6, Fig. 6) revealed no major morphological differences among treatments, although FBS-supplemented cultures exhibited a slightly slower progression of cytopathic effects compared with PRP-treated wells.

In BESc, extracellular viral kinetics resembled those observed in MDBK cells but with lower overall titers. At 24 hpi, the 5 % PRP condition produced a significant 1 log₁₀ reduction (*p* < 0.01) compared to 10 % FBS, and a 0.7 log₁₀ reduction relative to 10 % PRP. By 48 hpi, differences among treatments became non-significant (*p* > 0.05), while at 72 hpi, 5 % PRP again exhibited slightly lower titers (≈0.5 log₁₀, *p* < 0.01). Intracellularly, 10 % PRP consistently yielded lower viral titers than the FBS control—by approximately 0.5 log₁₀ at 48 hpi and 1 log₁₀ at 72 hpi (*p* < 0.01) (Fig. 5C and D). Representative micrographs at 48 hpi (Fig. 6E, F, Fig. 6, Fig. 6) confirmed typical BVDV-induced cytopathic changes, including vacuolization, but without clear morphological distinctions between treatment groups.

Collectively, these results show that PRP modulates BVDV replication in a time- and concentration-dependent manner. In MDBK cells, PRP transiently enhanced extracellular viral release during the early phase (24 hpi), whereas higher concentrations (10 %) subsequently suppressed replication. In BESc, both PRP concentrations exhibited modest inhibitory effects, particularly intracellularly at later time points, suggesting that PRP may restrict viral synthesis or assembly rather than release. These findings indicate a consistent antiviral tendency of PRP in primary endometrial cells and a biphasic modulation in permissive kidney-derived MDBK cells.

## 4 Discussion

Viruses such as BoAHV-1.1, BoGHV-4, and BVDV are major causes of bovine reproductive failure, including abortion and infertility (Atasever et al., 2023; Sheldon et al., 2009). Their mechanisms include uterine persistence and venereal transmission (BoAHV-1.1; (Graham, 2013). (Queiroz-Castro et al., 2019)), association with metritis (BoGHV-4; (Pérez et al., 2012; Verna, A. et al., 2008)), and delayed effects like embryonic loss (BVDV; (Beagley et al., 2010)). Conventional treatments, including systemic or intrauterine antibiotics, prostaglandin F₂α, or antiseptic solutions, mainly alleviate inflammation but do not prevent viral persistence and may adversely affect fertility (Lefebvre, 2022; Parikh et al., 2022; Runciman et al., 2009). Therefore, there is a pressing need for alternative therapeutic approaches.

PRP is a promising regenerative therapy due to its high concentration of growth factors that synergistically reduce inflammation and promote tissue repair (Li et al., 2019; Masuki et al., 2016). In this study, the effects of 5 % and 10 % PRP on the replication of these three bovine reproductive viruses were investigated in permissive MDBK cells (Marin et al., 2012; Morales-Aguilar et al., 2020; Romeo et al., 2021), and in bovine endometrial stromal cells (BESc) as a translational model. PRP modulated viral replication in a virus-, cell type-, and dose-related manner. In BoAHV-1.1-infected MDBK cells, 10 % PRP reduced extracellular viral release while increasing intracellular titers, suggesting interference with virion egress or late replication events. Conversely, in BESc, both intra- and extracellular titers decreased, indicating a broader antiviral effect potentially mediated by platelet-derived cytokines or growth factors that inhibit early viral gene expression. The apparent discrepancy observed with 5 % PRP treatment (an increase in extracellular viral titer (∼1 log) together with a marked decrease in intracellular titer (∼3 logs) at 48 hpi) likely reflects differential modulation of viral replication and release. PRP may enhance viral egress by altering membrane dynamics or stimulating extracellular vesicle (EV) release, while simultaneously restricting intracellular replication through platelet-derived antiviral factors or stress responses. Alternatively, this could represent a temporal uncoupling between intracellular assembly and extracellular release. Further analyses, including earlier time-point sampling, viral genome quantification by qPCR, and EV characterization, are planned to clarify whether PRP primarily promotes viral release or inhibits intracellular synthesis.

For BoGHV-4, PRP produced modest and variable effects: 5 % PRP reduced titers in MDBK cells at 48 hpi, whereas no consistent modulation was observed in BESc.

For BVDV, 10 % PRP transiently enhanced extracellular release during early infection but suppressed titers at later stages, particularly in BESc. These divergent responses underscore the complexity of PRP–virus–host interactions, likely influenced by viral genome type (DNA vs RNA) and cellular environment. Overall, PRP appears capable of restricting viral replication at specific stages, potentially through combined effects on cytokine signalling, oxidative balance, and membrane remodelling (Tebaldi et al., 2016), though variability across conditions highlights the need for further mechanistic studies integrating molecular and kinetic analyses. The main limitations of this work include the absence of platelet-poor plasma (PPP) controls, use of PRP from a single donor, testing of only two concentrations and one strain per virus, high confluence at infection (∼90 %), and the intrinsic constraints of *in vitro* models that cannot reproduce viral latency or persistence. Our research group recently reported that PRP modulates inflammatory and antiviral gene expression in bovine endometrial cells infected with BoGHV-4 and exposed to lipopolysaccharide (López et al., 2025), reinforcing the immunomodulatory role of PRP in bovine reproductive tissues. As mentioned, a standardized PRP preparation from a single donor heifer was employed to ensure experimental consistency, with technical replicates confirming internal reproducibility. However, as PRP is a biologically variable product influenced by donor factors, platelet concentration, and preparation methods (Alves et al., 2021; Dohan-Ehrenfest et al., 2009) future studies could expand on these findings by incorporating multiple donor-derived PRP samples, alongside platelet-poor plasma (PPP) controls, extended dose ranges, and complementary molecular assays assessing viral loads, cytokine expression, and antiviral gene responses to define the mechanisms underlying PRP’s modulatory effects. From a One Health perspective, improving reproductive efficiency in cattle contributes to food security, animal welfare, and rational antimicrobial use. PRP, being an autologous and low-risk biological product, represents a sustainable alternative aligned with these principles. In conclusion, this proof-of-concept study provides preliminary mechanistic evidence that PRP can modulate viral replication dynamics in bovine cells. Although preliminary, these findings establish a foundation for mechanistic and *in vivo* investigations aimed at validating PRP as a potential complementary therapy for viral reproductive disorders in cattle.

## 5 Conclusion

This study provides the first evidence that PRP can modulate the replication dynamics of bovine reproductive viruses *in vitro*. PRP displayed virus-, cell type-, and dose-related effects, suggesting that platelet-derived factors may interfere with distinct stages of viral replication, including intracellular accumulation and extracellular release. However, given the limitations of this exploratory work, the findings should be interpreted as preliminary mechanistic insights rather than direct indicators of clinical efficacy. Future studies incorporating platelet-poor plasma controls, multiple donors, additional viral strains, and *in vivo* models will be essential to clarify the biological relevance of these observations and to evaluate the potential of PRP as a supportive therapy for bovine reproductive viral infections.

## Funding sources

This work was supported by Agencia Nacional de Promoción Científica y Tecnológica through PICT Aplicado 2021–0039 and PICT 2020 Serie A-01,012

## Data statement

The data that support the findings of this study are available from the corresponding author upon reasonable request.

## CRediT authorship contribution statement

**Valentina Andreoli:** Writing – original draft, Methodology, Investigation. **Sofia Lopez:** Writing – original draft, Methodology, Investigation. **Santiago Germán Delgado:** Formal analysis, Data curation. **Sandra Elizabeth Pérez:** Writing – review & editing, Validation. **Susana Beatriz Pereyra:** Methodology. **Erika Analía Gonzalez Altamiranda:** Supervision. **Florencia Romeo:** Writing – review & editing, Supervision. **Stefano Grolli:** Supervision, Conceptualization. **Andrea Elizabeth Verna:** Resources, Project administration, Conceptualization.

## Declaration of competing interest

The authors declare that they have no known competing financial interests or personal relationships that could have appeared to influence the work reported in this paper.

## Data Availability

Data will be made available on request.

## Appendix Supplementary materials

Image, application 1

Image, application 2
