# Supplementary material for: Modulatory effects of platelet-rich plasma on viral kinetics of BoAHV-1.1, BoGHV-4, and BVDV in bovine cell cultures: A proof-of-concept study
Source: Virus Res. 2025 Oct 31;361:199653. doi: 10.1016/j.virusres.2025.199653 (PMC12634297; doi:10.1016/j.virusres.2025.199653)
Supplement: Supplementary file 1 [file mmc1.docx]

**Supplementary Figure S1.** Representative microphotographs of bovine endometrial stromal cells cultured at passages P0, P1 and P2. Cells maintained a typical fibroblast-like morphology and adherence throughout early passages, with no observable morphological alterations, senescence, or spontaneous differentiation.

40μm.


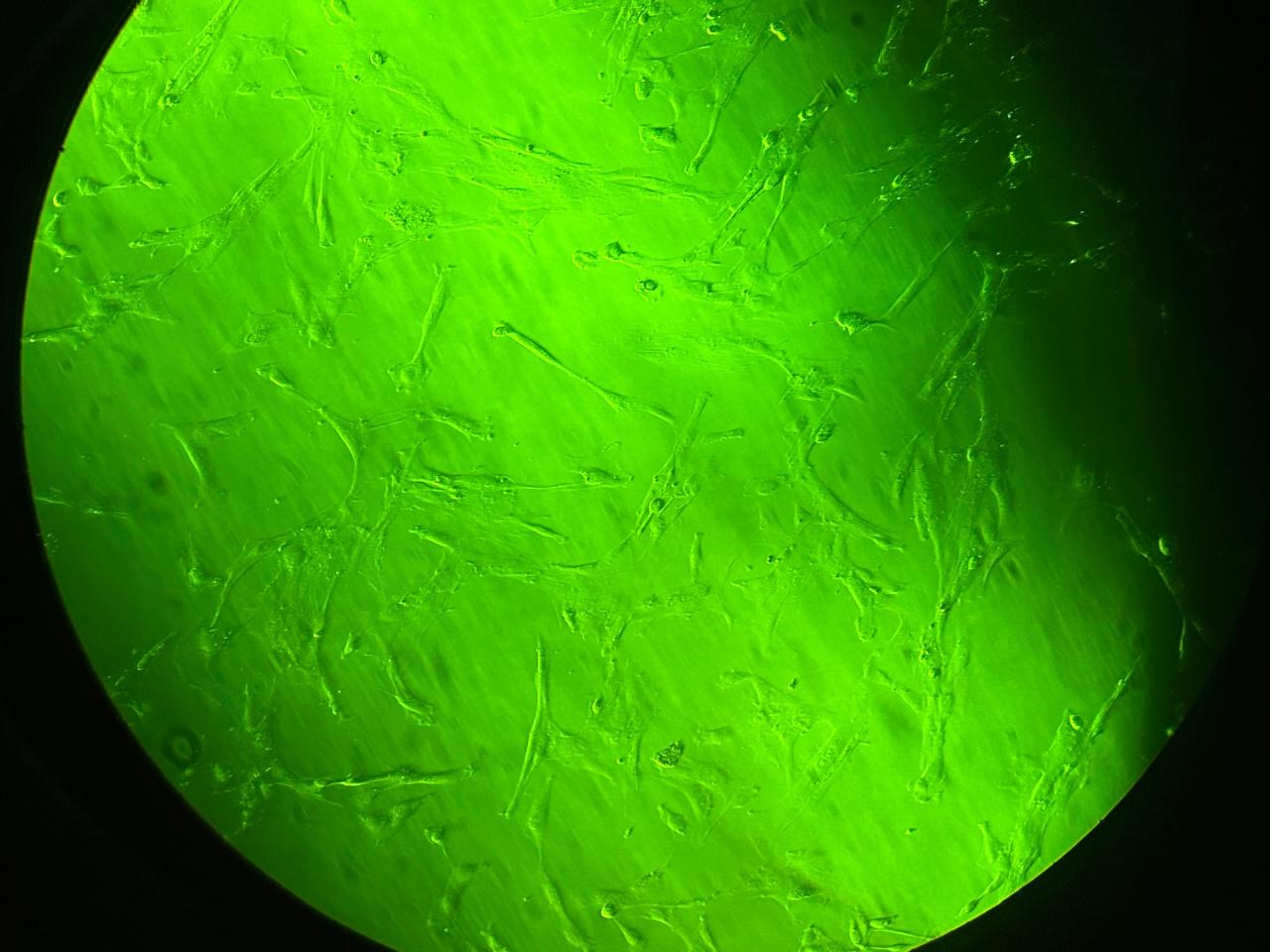


Passage 0


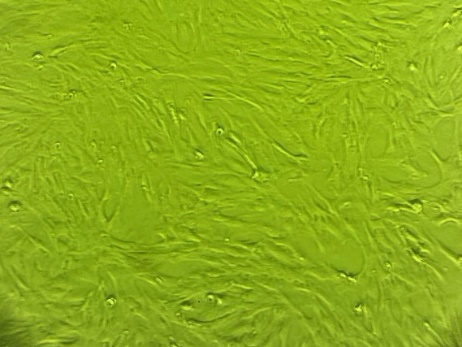


Passage 1


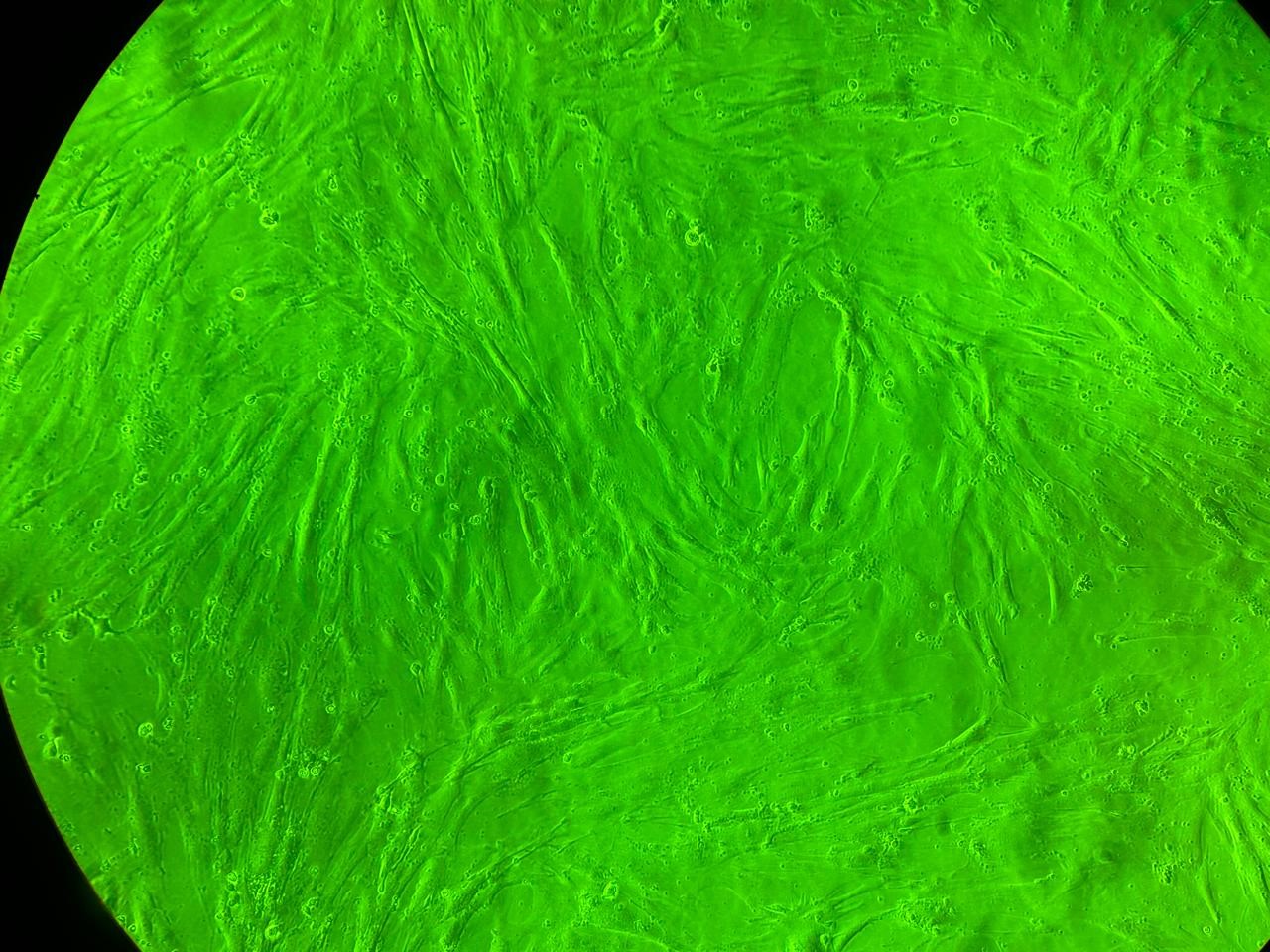


Passage 2
