# Supplementary material for: Modulatory effects of platelet-rich plasma on viral kinetics of BoAHV-1.1, BoGHV-4, and BVDV in bovine cell cultures: A proof-of-concept study
Source: Virus Res. 2025 Oct 31;361:199653. doi: 10.1016/j.virusres.2025.199653 (PMC12634297; doi:10.1016/j.virusres.2025.199653)
Supplement: Supplementary file 2 [file mmc2.docx]

***Supplementary Materials (Appendix A)***

*3.1.1. Replication Kinetics of BoAHV-1.1 in MDBK cell line*

| **MDBK CELL LINE** |  |  |  |  |  |  |  |
| --- | --- | --- | --- | --- | --- | --- | --- |
|  |  |  |  | **Hours post-infection (hpi)** | | |  |
|  |  |  |  |  |  |  |  |
|  | **BoAHV-1.1** |  | **TREATMENT** | **24** | **48** | **72** |  |
|  |  |  |  |  |  |  |  |
|  | Extracellular |  | 10% FBS | 5.25ᵃ±0.19 | 7.15ᵃ±0.14 | 7.25ᵃ±0.23 |  |
|  |  |  | 5% PRP | 5.08ᵃ±0.21 | 7.42ᵃ±0.15 | 7.16ᵃ±0.19 |  |
|  |  |  | 10% PRP | 4.89ᵃ±0.05 | 6.13^b^±0.35 | 7.20ᵃ±0.37 |  |
|  | Intracellular |  | 10% FBS | 5.83ᵃ±0.04 | 6.95ᵃ±0.06 | 6.91ᵃ±0.07 |  |
|  |  |  | 5% PRP | 5.80ᵃ±0.01 | 7.45ᵇ±0.10 | 6.95ᵃ±0.06 |  |
|  |  |  | 10% PRP | 5.80ᵃ±0.08 | 7.43ᵇ±0.20 | 7.03ᵃ±0.07 |  |
|  |  |  |  |  |  |  |  |

*Table S1:* Replication kinetics of BoAHV-1.1 in Madin-Darby bovine kidney (MDBK) cells. Viral titres (log₁₀ TCID₅₀/mL) are expressed as the mean ± SD of three independent replicates at indicated post-infection time points. Superscript letters within columns denote groups without statistically significant differences (LSD test, α = 0.05) between PRP (5% or 10%) and FBS treatments.

*3.1.2. Replication Kinetics of BoAHV-1.1 in BESc*

|  |  |  | **BESc** |  |  |  |  |
| --- | --- | --- | --- | --- | --- | --- | --- |
|  |  |  |  | **Hours post-infection (hpi)** | | |  |
|  |  |  |  |  |  |  |  |
|  | **BoAHV-1.1** |  | **TREATMENT** | **24** | **48** | **72** |  |
|  |  |  |  |  |  |  |  |
|  | Extracellular |  | 10% FBS | 5.33^a^±0.01 | 7.33^a^±0.03 | 7.76^a^±0.01 |  |
|  |  |  | 5% PRP | 5.66^a^±0.01 | 7.83ᵇ±0.03 | 7.66^a^±0.01 |  |
|  |  |  | 10% PRP | 4.90ᵇ±0.28 | 7.13^a^±0.01 | 7.83^a^±0.01 |  |
|  | Intracellular |  | 10% FBS | 6.83^a^±0.01 | 7.76^a^±0.03 | 7.76^a^±0.01 |  |
|  |  |  | 5% PRP | 6.73^a^±0.01 | 7.76^a^±0.03 | 7.76^a^±0.01 |  |
|  |  |  | 10% PRP | 6.53^a^±0.01 | 7ᵇ±0.28 | 7.13ᵇ±0.01 |  |
|  |  |  |  |  |  |  |  |

*Table S2*: Replication kinetics of BoAHV-1.1 in bovine endometrial stromal cells (BESc). Viral titres (log₁₀ TCID₅₀/mL) are expressed as the mean ± SD of three independent replicates at specified post-infection time points. Superscript letters within each column indicate groups without statistically significant differences (LSD test, α = 0.05) between PRP (5% or 10%) and FBS treatments.

*3.1.3. Replication Kinetics of BoGHV-4 in MDBK cell line*

|  |  |  |  |  |  |  |  |
| --- | --- | --- | --- | --- | --- | --- | --- |
|  |  |  |  | **Hours post-infection (hpi)** | | |  |
|  | **MDBK CELL LINE** |  |  |  |  |  |  |
|  | **BoGHV-4** |  | **TREATMENT** | **24** | **48** | **72** |  |
|  |  |  |  |  |  |  |  |
|  | Extracellular |  | 10% FBS | 2.90ᵃ±0.14 | 7.33ᵃ±0.01 | 6.43ᵃ±0.11 |  |
|  |  |  | 5% PRP | 4.13ᵇ±0.01 | 8.18ᵇ±0.49 | 7.50ᵇ±0.23 |  |
|  |  |  | 10% PRP | 3.33ᵃ±0.01 | 4.00^c^±0.21 | 7.79ᵇ±0.05 |  |
|  | Intracellular |  | 10% FBS | 4.66ᵃ±0.01 | 7.13ᵃ±0.03 | 8.02ᵃ±0.01 |  |
|  |  |  | 5% PRP | 4.42ᵃ±0.01 | 4.73ᵇ±0.03 | 7.66ᵃ±0.01 |  |
|  |  |  | 10% PRP | 5.33ᵇ±0.03 | 6.83^a^±0.01 | 7.71ᵃ±0.03 |  |
|  |  |  |  |  |  |  |  |

*Table S3:* *Replication kinetics of the BoGHV-4 in Madin-Darby bovine kidney (MDBK) cells. Viral titres are expressed as log₁₀ TCID₅₀/mL and represent the mean ± standard deviation of three independent replicates at the indicated post-infection time points. Superscript letters within each column indicate no statistically significant differences (LSD test, α = 0.05) between PRP treatments (5% or 10%) and FBS at each time point.*

*3.1.3. Replication Kinetics of BoGHV-4 in BESc*

|  |  |  |  |  |  |  |  |
| --- | --- | --- | --- | --- | --- | --- | --- |
|  |  |  |  | **Hours post-infection (hpi)** | | |  |
|  |  |  |  |  |  |  |  |
|  | **BoGHV-4** |  | **TREATMENT** | **24** | **48** | **72** |  |
|  |  |  |  |  |  |  |  |
|  | Extracellular |  | 10% FBS | 1.83^a^±0.04 | 2.13^a^±0.72 | 4.53^a^±0.04 |  |
|  |  |  | 5% PRP | 1.83^a^±0.01 | 2^a^±0.21 | 3.9^b^±0.14 |  |
|  |  |  | 10% PRP | 1.83^a^±0.007 | 1.83^a^±0.007 | 4.33^a^±0.03 |  |
|  | Intracellular |  | 10% FBS | 2.76^a^±0.01 | 4.53^a^±0.03 | 6.13^b^±0.01 |  |
|  |  |  | 5% PRP | 2.53^a^±0.01 | 4.13^a^±0.03 | 5.83^a^±0.01 |  |
|  |  |  | 10% PRP | 2.9^a^±0.03 | 4.53^a^±0.01 | 5.66^a^±0.03 |  |
|  |  |  |  |  |  |  |  |

*Table S4: Replication kinetics of the BoGHV-4 in bovine endometrial stromal cells (BESc). Viral titres are expressed as log₁₀ TCID₅₀/mL and represent the mean ± standard deviation of three independent replicates at the indicated post-infection time points. Superscript letters within each column indicate no statistically significant differences (LSD test, α = 0.05) between PRP treatments (5% or 10%) and FBS at each time point.*

*3.1.5. Replication Kinetics of BVDV in MDBK cell line*

|  | **MDBK CELL LINE** |  |  |  |  |  |  |
| --- | --- | --- | --- | --- | --- | --- | --- |
|  |  |  |  | **Hours post-infection (hpi)** | | |  |
|  |  |  |  |  |  |  |  |
|  | **BVDV** |  | **TREATMENT** | **24** | **48** | **72** |  |
|  |  |  |  |  |  |  |  |
|  | Extracellular |  | 10% FBS | 3.65ᵃ±0.25 | 5.80ᵃ±0.06 | 5.86ᵃ±0.08 |  |
|  |  |  | 5% PRP | 4.87ᵇ±0.01 | 5.76ᵃ±0.08 | 6.04ᵃ±0.07 |  |
|  |  |  | 10% PRP | 4.17^c^±0.16 | 5.30ᵇ±0.23 | 5.92ᵃ±0.15 |  |
|  | Intracellular |  | 10% FBS | 6.28ᵃ±0.69 | 6.92ᵃ±0.06 | 7.35ᵃ±0.19 |  |
|  |  |  | 5% PRP | 5.80ᵃ±0.06 | 6.80ᵃ±0.06 | 7.00ᵃ±0.06 |  |
|  |  |  | 10% PRP | 6.00ᵃ±0.06 | 6.83ᵃ±0.14 | 6.78ᵃ±0.03 |  |
|  |  |  |  |  |  |  |  |

Table S5: Replication kinetics of the BVDV in Madin-Darby bovine kidney (MDBK) cells. Viral titres are expressed as log₁₀ TCID₅₀/mL and represent the mean ± standard deviation of three independent replicates at the indicated post-infection time points. Superscript letters within each column indicate no statistically significant differences (LSD test, α = 0.05) between PRP treatments (5% or 10%) and FBS at each time point.

*3.1.6. Replication Kinetics of BVDV in BESc*

|  | **BESc** |  |  |  |  |  |  |
| --- | --- | --- | --- | --- | --- | --- | --- |
|  |  |  |  | **Hours post-infection (hpi)** | | |  |
|  |  |  |  |  |  |  |  |
|  | **BVDV** |  | **TREATMENT** | **24** | **48** | **72** |  |
|  |  |  |  |  |  |  |  |
|  | Extracellular |  | 10% FBS | 4.41^a^±0.59 | 4.92^a^±0.12 | 5.33^a^±0.03 |  |
|  |  |  | 5% PRP | 3.44^b^±0.62 | 5.33^a^±0.01 | 4.9^b^±0.28 |  |
|  |  |  | 10% PRP | 4^a^±0.14 | 5.05^a^±0.07 | 5.33^a^±0.01 |  |
|  | Intracellular |  | 10% FBS | 5.33^a^±0.1 | 5.73^a^±0.1 | 5.67^b^±0.19 |  |
|  |  |  | 5% PRP | 5^a^±0.01 | 5.67^a^±0.23 | 4.9^a^±0.28 |  |
|  |  |  | 10% PRP | 5.13^a^±0.01 | 5.13^b^±0.03 | 4.85^a^±0.03 |  |
|  |  |  |  |  |  |  |  |

*Table S6:* Replication kinetics of the BVDV in bovine endometrial stromal cells (BESc). Viral titres are expressed as log₁₀ TCID₅₀/mL and represent the mean ± standard deviation of three independent replicates at the indicated post-infection time points. Superscript letters within each column indicate no statistically significant differences (LSD test, α = 0.05) between PRP treatments (5% or 10%) and FBS at each time point.
